# Diagnosis and Management of Keratoconus—A Narrative Review of Clinicians’ Perspectives

**DOI:** 10.3390/children9121973

**Published:** 2022-12-15

**Authors:** Minji Song, Tanya Chen, Adam Moktar, Elsie Chan, Elaine W Chong, Mark Daniell, Srujana Sahebjada

**Affiliations:** 1 Centre for Eye Research Australia, Melbourne, VIC 3002, Australia; 2 Ophthalmology, Department of Surgery, The University of Melbourne, Melbourne, VIC 3010, Australia; 3 Royal Victorian Eye and Ear Hospital, Melbourne, VIC 3002, Australia

**Keywords:** keratoconus, diagnosis, management, treatment, attitude, practices, opinion, optometrist, ophthalmologist

## Abstract

This review discusses the current practices, attitudes, and trends in diagnosing and managing keratoconus (KC) in adults and children by optometrists and ophthalmologists in order to highlight the differences on a global scale. Two independent reviewers searched the electronic databases and grey literature for all potential articles published from 1 January 2000 to 1 June 2022 on management of KC. Keywords used in searches included “keratoconus”, “diagnosis”, “management”, “treatment”, “attitude”, “practices”, “opinion”, “optometrist”, “ophthalmologist”, “consensus”, and “protocol”. A total of 19 articles was included in this review—12 from the database search and seven from the grey literature. Although a common stepwise approach of non-surgical management was noted, there were differences in the rates of prescribing rigid gas permeable lenses. Furthermore, while clinicians agreed on the need for early diagnosis, the timeline and type of referral varied significantly. A similar discordance was found in the milestones for surgical intervention and preferred surgical techniques. Practice patterns in keratoconus diagnosis and management vary throughout the world. Multiple recommendations and suggestions to minimise the differences have been provided in the literature, with the main themes being improvement in education, interdisciplinary patient care, and further research to reach consensus.

## 1. Introduction

Keratoconus (KC) is bilateral and usually asymmetric corneal ectasia, whose etiology is still under study, but probably includes both genetic predisposition and environmental factors, such as eye rubbing and nocturnal ocular compression [1]. It typically affects young adults [2]; however, there are increasing reports of KC in children [3]. It is characterised by progressive conical distortion and stromal thinning leading to apical protrusion, irregular astigmatism, and significant impairment of visual acuity [4]. The diagnosis of KC is reliant on comprehensive history taking and corneal assessment, involving slit-lamp and corneal topography, to identify these salient features [5]. In addition, it can lead to complications, such as corneal hydrops, and require corneal transplantation, although recently a decreasing tendency has been observed in KC as an indication for keratoplasty, probably related to the availability of corneal crosslinking [1,6,7].

KC is the most common type of corneal ectasia, with global epidemiological data estimating a prevalence between 0.2 and 4790 per 100,000 persons, and an incidence between 1.5 and 25 cases per 100,000 persons per year [4]. Although the disease can affect young children, with the youngest documented case of KC being at age 4 [8], KC typically develops in the second and third decades of life and stabilises by the fourth decade [4].

In general, the management of keratoconus is dependent on disease progression and severity [4]. In early stages, spectacles and soft contact lenses might suffice in managing changes in visual function [4]. However, as the disease progresses, and irregular astigmatism develops, rigid contact lenses might be necessary as they provide neutralisation of the corneal irregularity by the tear lens [4]. In more advanced cases, scleral lenses might be more beneficial in neutralising the irregular cornea, and corneal surgery might be considered [4]. To prevent progression of the disease, a corneal crosslinking (CXL) procedure might be proposed for patients with sufficient corneal thickness (greater than 400 micrometres) [9].

However, given the increasing number of paediatric KC cases, and the comparatively scarce literature on management of paediatric KC, the above protocols were mostly derived based on studies exploring KC management in adults [4]. From what is known, management of KC in the paediatric population can differ from the standard protocols described above, owing to the structural and behavioural differences between children and adults [3]. Moreover, disease progression in paediatric KC is far more aggressive than in adults [10] and therefore requires closer follow-up protocols and earlier consideration of interventions, such as CXL, that aim to halt progression [3,9].

To better understand the differences between management of KC in the paediatric and adult population, this review will explore the approaches and attitudes of optometrists and ophthalmologists in diagnosing and managing KC in adults and children. A focus will be made on the diagnosis, non-surgical-method referral patterns, and surgery preferences in these groups.

## 2. Materials and Methods

### 2.1. Literature Search Strategy

Electronic databases, including Scopus, Web of Science, PubMed, and Cochrane CENTRAL, were searched for all potential articles published from 1 January 2000 to 1 June 2022. MeSH terms and keywords used to balance the sensitivity and specificity of the search included “keratoconus”, “diagnosis”, “management”, “treatment”, “attitude”, “practices”, “opinion”, “optometrist”, “ophthalmologist”, “consensus”, and “protocol”. The same keywords were used for the grey literature search. This review was carried out according to the guidelines of the Declaration of Helsinki.

### 2.2. Inclusion Criteria

Primary studies were considered eligible for inclusion in this review if they met the following criteria:

- Full original articles
- Published from 1 January 2000 to 1 June 2022
- English language only
- Studies involving human beings only
- Randomised controlled trials and non-randomised observational studies (cohort, case-control, and cross-sectional studies)
- For the grey literature search, educational material for optometrists or ophthalmologists and articles written by optometrists or ophthalmologists were included.

There was no limit on the population group in terms of age, sex, ethnicity, or co-morbidities.

### 2.3. Exclusion Criteria

- Studies that explored patients’ attitudes, not that of clinicians, towards keratoconus management
- Review articles, case reports, surveys, PowerPoint presentations, abstract-only studies, and studies without full text available were excluded

### 2.4. Study Selection

Two authors (Chen, Song) independently screened the titles of the publications for the inclusion and exclusion criteria and all potential studies were noted. The titles and abstracts were read to further filter the included studies. The complete texts of the studies were then obtained and read in full to fulfil the final inclusion. Any disagreement was resolved by reaching a consensus through discussion.

### 2.5. Data Extraction

Two authors (Chen, Song) independently extracted information from the included studies. Data extracted included the title, authors, date, country of origin, study design, demographics, sample size, disease definition, diagnosis, and management practices of optometrists and ophthalmologists. Any disagreement was resolved by reaching a consensus through discussion.

### 2.6. Outcome Measures

The outcome of the study was current keratoconus management patterns, attitudes and practices of optometrists and ophthalmologists regarding diagnosis, non-surgical methods, referrals to ophthalmologists, and surgical methods. This included the mode and timing of management, and any potential barriers limiting comprehensive care.

## 3. Results

**Figure 1:**
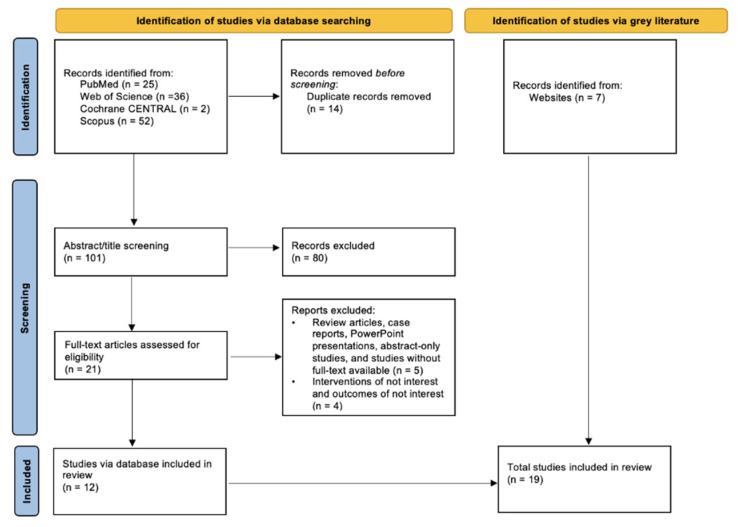
PRISMA Diagram.

Identification of studies involved two arms: database searching and grey literature. Through database searching, a total of 115 potentially eligible records were extracted in the initial retrieval process. During the screening, 14 records were eliminated due to duplication, and 80 were eliminated based on the study title and abstract. Of the 21 full-text articles reviewed, 12 articles were excluded for not meeting the inclusion criteria. Along with the seven studies from grey literature, a total of 19 studies was included in the review. The process used to search and identify studies is illustrated in Figure 1.

### 3.1. Characteristics of Included Studies

**Figure 2:**
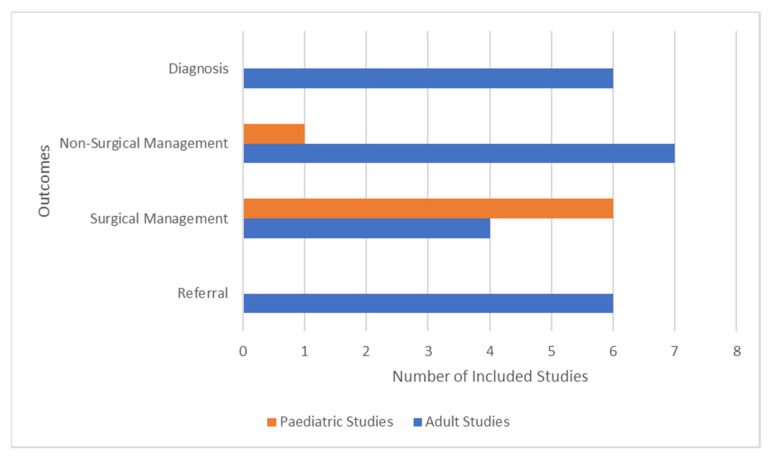
Bar chart characterizing the number of included paediatric (orange) and adult (blue) studies that explored diagnosis, non-surgical management, surgical management, and referral as their key outcomes.

There were six studies that explored specifically paediatric patients, while the remaining studies investigated practices of clinicians. Of the studies focusing on paediatric keratoconus, there were two based in Switzerland [11,12], one in Italy [13], one in India [14], one in the Netherlands [15] and two unknown [16,17]. Studies observed practices of ophthalmologists from Switzerland [18], South Korea [19], and Australia [20,21], and optometrists from Australia [22], Latin American countries [23], US [24,25,26], UK and Spain [27] as well as two studies involving panellists of ophthalmologists around the world [28,29]. Four studies utilised a format of questionnaire or survey to gain insight into clinicians’ various practices or medical knowledge [18,22,23,27]. The two studies that involved panellists of clinicians generated agreements in management through discussion [28,29]. One study retrospectively identified the outcome through patient data [19]. There were two articles outlining the clinician’s individual management practice targeted at optometric [24] or ophthalmic professionals [21], and three education courses for other optometrists [25,26] or ophthalmologists [20]. This is summarised in Table 1 and Figure 2.

**Figure 3:**
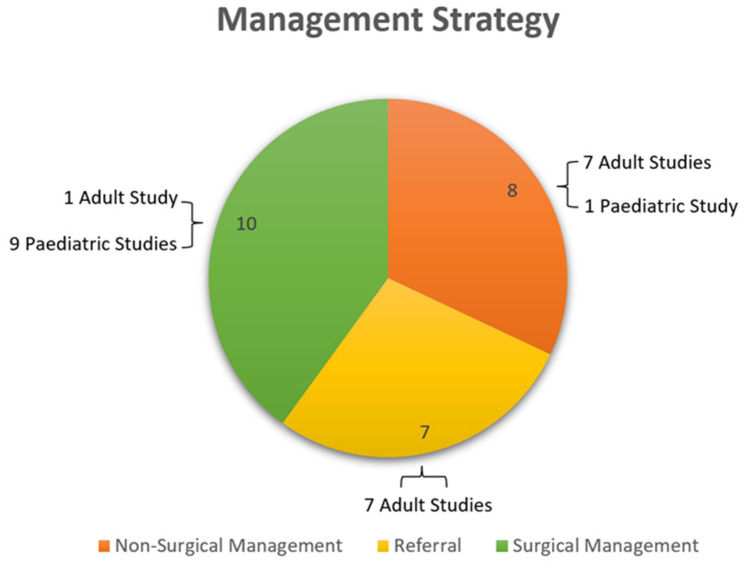
Pie chart illustrating the number of included studies (further broken down into pediatric and adult studies) that focused on non-surgical management (orange), referral (yellow), and surgical management (green) as a management strategy for keratoconus.

Various patterns of keratoconus diagnosis and management through non-surgical methods, referral patterns, and surgical methods could be seen. Figure 3 illustrates the number of studies that focussed on non-surgical management, referral, and surgical management. Findings from this study stipulate for further large-scale longitudinal research to illuminate other differences and formulate ways to reach a consensus both in theory and in clinical settings.

### 3.2. Diagnosis

The studies indicated a significant difference in the rates of KC diagnoses across the world. The majority of respondents in the UK (65.1%) and Spain (65.7%) reported fewer than five cases of keratoconus detected per year [27]. Notably, Ortiz-Toquero and Martin [27] were unable to find statistical significance for this. A similar rate of diagnosis was evident in European optometrists (75.7%); however, only 44.4% of Latin American optometrists responded in concordance with this low rate of diagnosis [23].

Despite the variance in the rate of diagnosis, the literature details quite consistent diagnostic criteria for keratoconus. Optometrists and ophthalmologists agreed on the need for early diagnosis [20,25,29] and raised that a combination of factors is often employed for keratoconus diagnosis. These include the use of a positive family history for keratoconus, visual acuity scissor shadows in retinoscopy, corneal topography, and slit lamp signs for diagnosis [20,23,25,27]. The literature strongly emphasized the importance of early diagnosis of KC in pediatric patients due to faster disease progression [14]. Practitioners in Latin America and Europe utilize a severity-classification system such as Amsler–Krumeich [23,27]. However, Gomes et al.’s [28] panel agreed that the Amsler–Krumeich classification system was inadequate and failed to accommodate current advances in keratoconus knowledge and technology.

### 3.3. Management Regarding the Attitudes of Primary Eye Care Physicians

#### 3.3.1. Non-Surgical Methods

Various practices involving non-surgical methods of treating keratoconus were portrayed in the studies. There was a strong agreement that the most important goals of non-surgical methods in managing keratoconus are to halt disease progression and provide visual rehabilitation [20,25,28,29]. Ophthalmologists emphasised verbal guidance to patients regarding the importance of not rubbing the eyes as the most important non-surgical method [28,29]. Studies reported a stepwise approach to optical correction, moving to the next option if one fails: starting from glasses, soft contact lenses, and rigid lenses (with the preferred one for keratoconus being gas-permeable lenses), then to specialised keratoconus contact lenses, such as hybrid, piggy-back, scleral and miniscleral [20,28,29]. It was agreed that although contact lenses provide visual rehabilitation, they do not halt progression [20,28,29]. Studies also suggested contact lenses to provide improved visual acuity after surgical treatment to stabilise the cornea; Bhatt [20] mentioned Rigid Gas Permeable (RGP) contact lenses (CL) while Ibach [25] recommended specialty lenses.

There was one study conducted in India observing management of specifically paediatric KC patients. CLs, mostly Rose K2 type, were dispensed to 12.1% (n  =  14), and glasses to 20.7% (n  =  24) of patients, without any additional intervention [14]. Both management methods demonstrated favourable outcomes with no significant changes in best corrected visual acuity (BCVA), uncorrected visual acuity and astigmatism at 12- and 24-month follow-up. After 24 months of CL use, there was an improvement in visual acuity from baseline mean BCVA of 0.23  ±  0.22 logMAR to 0.17  ±  0.6 logMAR (*p* = 0.001).

Studies demonstrate differences in rates of prescribing contact lenses. Hodge and Chan’s [22] study explored Australian optometrists and 35.4% of the survey participants reported that they prescribe soft contact lenses to KC patients daily. For gas-permeable lenses, 9.2% of optometrists prescribed them daily while 47.7% prescribed them at least once a month. In comparison, 54.8% of UK optometrists and 28.1% of those in Spain prescribed them at least once per month [27].

Difficulty in prescribing rigid contact lenses was reported in multiple studies. A majority of optometrists from Latin America (74.0%), the UK (67.5%), and Spain (70.7%) responded that fitting is more difficult in keratoconus eyes. This is in accordance with Hodge and Chan’s [22] study, which also outlined the main barriers to prescribing for Australian optometrists as a lack of experience with fitting RGP lenses, time taken, and low market demand. Denny [29] also reported that ophthalmologists in the US have seen a downward trend in fitting not only RGP lenses but also keratoconus-specialised lenses due to a lack of training, substantial time commitment, or relatively low reimbursement. Due to a variety of barriers, there is a discrepancy in whether optometrists think training would help them increase rates of fitting RGP lenses. While some optometrists state that they would with more training (54% for Latin America, 25% for UK and Spain), others disagree (50% of optometrists in UK and Spain) [23,27]. Optometrists in Australia with greater experience were more likely to prescribe RGP lenses [22]. A common positive factor for increasing RGP prescription was access to corneal topography, noted in Australian and Latin American optometrist practices [18,22].

Differences in the level of knowledge of keratoconus management were also noted. Baenninger et al.’s [18] study investigated the level of keratoconus knowledge in Swiss ophthalmologists and observed a substantial mismatch from the expectations. Only 81% correctly recalled rigid contact lenses as one of the treatment modalities, and glasses was only reported by 20%.

#### 3.3.2. Referral Patterns

Across the literature it was found that patterns of referral differed based on the optometrist’s preference for contact lens management or surgical. This is most likely related to a lack of global consensus on the management of keratoconus by clinicians, which translates to variable referral patterns [25]. There were no studies which investigated the referral patterns amongst optometrists regarding keratoconus management in paediatric cohorts. A concordant idea shared by many of the education courses for optometrists highlighted the importance of early diagnosis and referral to a contact lens or corneal specialist for adults [20,24,25,26,27]. While all authors emphasised the significance of early diagnosis to prevent further vision damage, Chou [26] proposed that referral to a contact lens specialist is the most adequate approach for most keratoconus patients. Ajamian’s [24] interview shared a similar viewpoint, expressing that in most mild keratoconus cases, spectacle- or contact-lens correction is sufficient. Both Chou [26] and Ajamian [24] agreed that referral to a corneal specialist is only appropriate “when you no longer have anything to offer the patient” [24]. Ibach [25] instead put forth that referral to a corneal specialist for corneal crosslinking, then subsequent specialty contact lens management, provides the most improved long-term visual acuity. Huynh’s [21] preference was to refer all patients to a corneal specialist regardless of keratoconus severity once a diagnosis is made. Huynh [21] postulated that with the current emergence of KC treatments, practitioners must assist patients in making the most informed choices about their care.

A survey conducted by Hodge and Chan [22] amongst 71 optometrists in Australia held similar recommendations, as they found an absence of routine guidelines for referrals to ophthalmologists within their cohort. Consensus on a timeline for ophthalmologist referral for further management was not found, as respondents would vary in referral time from immediate to after signs of visual acuity degradation have been detected. While in the Hodge and Chan [22] survey, many respondents would refer if a patient’s visual acuity was between 6/9 to 6/12, another study [23] found that only a small number of Latin American optometrists would refer based on visual acuity. This irregularity among optometrists was also reported by Ortiz-Toquero and Martin [27] who conducted a survey among optometrists in the UK and Spain. Low global rates of referral to another optometrist for contact-lens fitting before an ophthalmologist for surgical interventions was also found [22,23,27].

### 3.4. Management Regarding the Attitudes of Opthalmologists

Practitioners generally agreed on the type of surgical management to be used in patients. Studies stated that CXL is utilised for corneal stabilisation and is the most commonly performed surgery for patients [25,28,29]. Primary eyecare providers in Gomes’ panel strongly recommended the use of CXL for young patients, even if satisfactory vision was achieved with glasses. This consensus was obtained after panellists were presented with two case scenarios of 15-year-old patients with either stable or progressive KC. Denny [29] and Gomes and Tan [28] also proposed deep anterior lamellar keratoplasty (DALK) and penetrating keratoplasty (PK) as techniques for improving visual acuity. Panellists in Gomes and Tan’s [28] and Denny’s [29] interviews concurred in their use of DALK predominantly for patients who display contact-lens intolerance, and use of PK for patients who had significant corneal scarring and very thin corneas. Clinicians in both the literatures agreed that descemetic baring DALK is the current gold standard in corneal transplantation. However, clinicians in the Denny [29] panel expressed that the technique is more surgically challenging and is performed at low rates in the US (2%). Denny [29] proposed this as a contributing factor to the panellists’ preference and use of PK over DALK as a surgical intervention. In contrast, experts in the Gomes and Tan [28] interview held a preference for DALK unless explicit indicators for PK were found. In particular, DALK with big bubble technique was attempted in more than 51% of patients.

There were two studies concerning the efficacy of surgical techniques other than CXL for paediatric patients. Feizi and Javadi [16] conducted a retrospective time-series study and found no significant difference in efficacy between PK and DALK in paediatric keratoconus. Both Feizi and Javadi [16] and Gupta and Saxena [14] found DALK to be advantageous over PK due to its decreased risk of allograft rejection and increased graft longevity. Researchers both reported this to be particularly beneficial in paediatric patients who require the grafts for longer than adult patients. The included paediatric CXL studies all reported positive outcomes up to 36 months following crosslinking surgery. Soeters and van der Valk [15] further found greater visual improvement in paediatric patients after CXL than adult patients.

The literature indicated a discordance in practitioners’ agreement on surgical management type and the stage at which it should be indicated. In Denny’s [29] interview, the panellists agreed that surgical interventions are considered when contact-lens use becomes intolerable for the patient. Panellists defined this as an inability to tolerate contact lenses for more than an hour. Panellists in Gomes and Tan [28] agreed that CXL should be performed in all corneal ectasia patients who display evidence of clinical progression, regardless of the patient’s age. They were unable to offer any consensus on the use of surgical interventions for patients without any evidence of clinical progression. Ibach [25] similarly recommended that CXL is appropriate for any patient showing signs of clinical progression to corneal ectasia. Furthermore, Ibach [25] agreed that despite the ‘relative contraindications’ for patients under 14 or over 65 years old, CXL is very effective with a low rate of adverse outcomes. Unlike the panellists in Denny’s [29] interview, Ibach [25] proposed that corneal crosslinking should be performed before specialty contact-lens use.

A failure to define a consistent timeline for surgical management was also found in studies investigating CXL for paediatric patients. Retrospective and prospective paediatric cohort studies report that CXL was performed if clinical progression was detected within a 1-to-3-month follow-up period [12,13,15], within a 6-month period [14,17], and within a 12-month period [11]. The inclusion criteria for clinical progression varied by study (Table 2).

## 4. Discussion

Practice patterns in keratoconus diagnosis and management by optometrists and ophthalmologists vary throughout the world. The literature suggested that while clinicians agree on the need for early diagnosis, the classification systems utilised for diagnosis vary by country [20,23,25,27,29]. In addition, the next steps after diagnosis are globally unclear. Evaluation of the differing rates of KC rates by country suggested that European optometrists annually diagnosed a lower number of cases than those in Latin America [23,27]. It must be noted however that these rates were self-reported in both studies and may not reflect actual frequencies of diagnosis. Nevertheless, the possible variation in KC diagnosis rates by country likely contribute to the differences in reported management patterns. Managing KC through non-surgical methods generally showed an agreement in the aim and indication to provide visual rehabilitation for patients, but there were significant differences in the specificities of clinical practice, mainly the type and rates of CLs to prescribe. Multiple factors explaining the differences were also reported by the studies and were mainly due to the different barriers to contact lens fitting [22,29]. Differences were also found in primary eyecare providers’ attitudes towards referring patients to corneal specialists and ophthalmologists [20,22,23,24,25,26,27]. The variance in recommended referral patterns suggests that there is an inadequate level of consistency between optometrists, past the point of diagnosis. It appears that there is a concordant belief that CXL should be performed in paediatric patients [12,13,15,25,28]; however, a consistent timeline or criteria for when surgical management should be enacted could not be established. This may perhaps be attributed to the relatively recent innovation of CXL in 2004. Such differences may also be the reasons for the existence of multiple training modules for clinicians in the grey literature [20,25,26]. However, as the articles in the grey literature also show discrepancies, this poses a further issue in reaching a consensus.

### 4.1. Clinical Implications

In terms of initial management, a stepwise approach to optical correction and corneal rehabilitation could be beneficial for patients, starting from glasses, soft contact lenses, and rigid lenses, with the preferred one for keratoconus being gas-permeable lenses, then to specialised keratoconus contact lenses, such as hybrid, piggy-back, scleral, and miniscleral [20,28,29].

The education courses for optometrists [20,24,25,26] reflected the disagreement in referral patterns to a corneal specialist versus contact lens specialist, initially found in the studies involving optometric attitudes to referral during clinical practice [22,27]. A consensus amongst the included studies could also not be obtained for a referral timeline [22,23,27]. The existing literature suggests that CXL is the preferred surgical method for ophthalmologists, particularly for paediatric patients due to their more aggressive progression of disease [14,15,16,25,28,29]. After surgery, RGP or KC specialty lenses could be considered for corneal stabilisation [20,25].

### 4.2. Recommendations for Research and Practice

Multiple recommendations and suggestions to reduce the differences in clinical practice have been provided by the literature, with the main themes being improvement in education, interdisciplinary patient care, and further research to reach consensus. There is a need for better general optometrist and ophthalmologist training, including RGP fitting, timing of ophthalmologist referral and DALK surgical technique to ensure a consistent global expertise in utilising the gold standard in corneal transplantation [22,23,27]. Substantial improvement in collaboration between clinicians, such as through international consortia, is necessary to devise a global classification and management system, especially for paediatric KC, and promote early diagnosis and optimal management [18]. In terms of research, there is a lack of studies investigating paediatric KC specifically, and an exploration of surgical techniques will assist optometrists in clarifying exact diagnostic criteria, or a timeline for surgical referral and enactment of surgical intervention [29]. There is an ongoing need for studies to track progress and evaluate clinicians’ knowledge and practices of managing KC patients.

### 4.3. Limitations of the Review

An important limitation of the included studies is that overall, there is a wide variety of outcomes investigated, such as the types of different management methods, the rates of a specific method being used, and the timing of the method taking place. This is not ideal when comparing results of different studies, but each study still provides important information about the outcomes. Furthermore, five included studies were limited to the attitudes and opinions of a single practitioner each [20,21,24,25,26]. While effective in their provision of a thorough and detailed attitude towards keratoconus management, the studies may not be generalised to the wider clinician population.

## 5. Conclusions

Practice patterns in KC diagnosis and management vary throughout the world, with differences arising from personal preferences of clinicians and various barriers to a certain management method. The discrepancies were mostly in the rates of prescribing rigid gas permeable lenses, and the timing or type of referral, leading to varied indications for surgical intervention.

## Figures and Tables

**Table 1:** Main characteristics of included studies.

Study	Country	Study Design	Sample Characteristics	Outcomes
Ajamian et al. [24]	US	Magazine article	Optometrist, n = 1	Referral
Baenninger et al. [18]	Switzerland	Cross-sectional study	Ophthalmologists, n = 100	Clinicians’ knowledge regarding keratoconus
Bhatt et al. [20]	Australia	Education course	Ophthalmologist, n = 1	Diagnosis, referral, non-surgical and surgical methods
Caporossi et al. [13]	Italy	Prospective non-randomized trial	KC (keratoconus) paediatric patients, n = 152	Surgical methods
Chatzis, N. and Hafezi, F. [11]	Switzerland	Retrospective time series study	KC paediatric patients, n = 26	Surgical methods
Chou et al. [26]	US	Education course	Optometrist, n = 1	Referral
Denny et al. [29]	Global	Magazine article	Ophthalmologists, n = 45	Diagnosis, non-surgical and surgical methods
Feizi et al. [16]	N/A	Retrospective time-series study	KC paediatric patients, n = 99	Surgical methods
Gomes et al. [28]	Global	Guideline	Ophthalmologists, n = 36	Diagnosis, non-surgical and surgical methods
Gupta et al. [14]	India	Cohort study	62 paediatric patients	Non-surgical methods, surgical methods
Hodge et al. [23]	Australia	Cross-sectional study	Optometrists, n = 71	Non-surgical methods, referral
Huynh et al. [21]	Australia	Article	Ophthalmologist, n = 1	Referral
Hwang et al. [19]	South Korea	Cohort study	KC patients, n = 10,612	Incidence of corneal transplantation
Ibach M. [25]	US	Education course	Optometrist, n = 1	Diagnosis, non-surgical methods
Ortiz-Toquero, S. and Martin, R. [27]	UK and Spain	Cross-sectional study	Optometrist, n = 464	Diagnosis, non-surgical methods, referral
Soeters, N. and van der Valk, R. [15]	Netherlands	Prospective non-randomized trial	KC patients, n = 95	Surgical methods
Vieira et al. [23]	Latin America	Cross-sectional study	Optometrists, n = 977	Diagnosis, non-surgical methods, referral
Vinciguerra et al. [12]	Switzerland	Prospective non-randomized trial	KC paediatric patients, n = 40	Surgical methods
Zotta et al. [17]	N/A	Retrospective time-series study	KC paediatric patients, n = 4	Surgical methods

**Table 2:** The inclusion criteria to determine clinical progression by study.

Study	Visual Acuity	Refraction Δ	Keratometry Δ	Topographic Surface Asymmetry Index Δ	Corneal Thickness
Caporossi, et al. [13]	Deterioration ≥ 1 Snellen line	Δ Sphere/Cylinder > 0.5 D	ΔKaverage > 0.5 D	Δ > 0.5 D	Reduction at thinnest point ≥ 10 μm
Chatzis, N. and Hafezi, F. [11]	N/A	N/A	ΔKmax > 1 D	N/A	N/A
Gupta, et al. [14]	N/A	N/A	Progressive steepening	N/A	Progressive thinning
Soeters, N. and van der Valk, R. [15]	Inclusion criteria were not delineated into subcategories				
Vinciguerra, et al. [12]	N/A	Δ Sphere/Cylinder > 3 D	ΔKaverage ≥ 1.5 D	N/A	≥400 μm at thinnest point after epithelial removal
Zotta, et al. [17]	N/A	Δ Spherical equivalent > 0.75 D	ΔKmax > 0.75 D	N/A	≥400 μm at thinnest point after epithelial removal

## Data Availability

Not applicable.
